# Revisiting synthetic lethality of Gcn5-related N-acetyltransferase (GNAT) family mutations in *Haloferax volcanii*

**DOI:** 10.1128/spectrum.01229-25

**Published:** 2025-07-02

**Authors:** Katherine R. Weber, Brianna Novillo, Julie A. Maupin-Furlow

**Affiliations:** 1 Department of Microbiology and Cell Science, Institute of Food and Agricultural Science, University of Florida 209721, Gainesville, Florida, USA; 2 Genetics Institute, University of Florida 145773, Gainesville, Florida, USA; University of Minnesota Twin Cities, St. Paul, Minnesota, USA

**Keywords:** archaea, post-translational modification, lysine acetyltransferase, mutant generation, *Haloferax volcanii*

## Abstract

Lysine acetylation is a post-translational modification that occurs in all domains of life, highlighting its evolutionary significance. Previous genome comparison identified three Gcn5-related N-acetyltransferase (GNAT) family members as lysine acetyltransferase homologs (Pat1, Pat2, and Elp3) and two deacetylase homologs (Sir2 and HdaI) in the halophilic archaeon *Haloferax volcanii*, with *elp3* and *pat2* proposed as a synthetic lethal gene pair. Here, we advance these findings by performing single and double mutagenesis of *elp3* with the *pat1* and *pat2* lysine acetyltransferase gene homologs. Genome sequencing and PCR screens of these strains reveal successful generation of Δ*elp3,* Δ*pat1*Δ*elp3*, and Δ*pat2*Δ*elp3* mutant strains. Although these mutant strains exhibited a reduced growth rate compared to the parent, they remained viable. Overall, this study provides genetic evidence that *elp3* and *pat2*, while impacting cell growth, are not a synthetic lethal gene pair as previously reported.

**IMPORTANCE:**

Here, we reveal by whole-genome sequencing that the GNAT family gene homologs *elp3* and *pat2* can be deleted in the same *Haloferax volcanii* strain. Beyond the targeted deletions, minimal differences between the parent and Δ*elp3*Δ*pat2* mutant were observed, suggesting that suppressor mutations are not responsible for our ability to generate this double mutant strain. Elp3 and Pat2, thus, may not share as close a functional relationship as implied by earlier study. Our finding is significant as Elp3 is thought to function in acetylation in tRNA modification, while Pat2 likely functions in the lysine acetylation of proteins.

## INTRODUCTION

Post-translational modifications (PTMs) are chemical modifications that occur after protein synthesis, influencing the charge, structure, and function of proteins (1). Among PTMs, lysine acetylation is considered an ancient form that is evolutionarily conserved in all domains of life (2). It regulates key biological processes, including metabolism and chromatin structure, and is often responsive to external stimuli such as nutrient availability and oxidative stress (3–5). Lysine acetylation (KAc) involves the transfer of an acetyl group from the metabolic intermediates acetyl-coenzyme A (acetyl-CoA) or acetyl-phosphate (acetyl-P) to the lysine residue of a target protein (6). This reaction can occur non-enzymatically or enzymatically by lysine acetyltransferases, with lysine deacetylases catalyzing the removal of the acetyl group, offering opportunities for regulatory control (3). While research has examined lysine acetylation extensively in the context of histone modification and bacterial metabolism (3–5), the role of this PTM in archaeal cell biology is less studied.

*Haloferax volcanii* (*Hv*) is a hypersaline-adapted archaeon that tolerates harsh environmental conditions such as ultraviolet irradiation (7), desiccation (8), extreme temperatures and pH (9, 10), heavy metals (11), and strong oxidants (12, 13). Proteomic and genetic studies of *H. volcanii* reveal a correlation between oxidative stress and lysine acetylation (12–14). Previous genome comparison identified three histone lysine acetyltransferase (HAT) homologs of the Gcn5-related N-acetyltransferase (GNAT) family, Pat1, Pat2, and Elp3, and two histone deacetylase (HDAC) homologs including the NAD^+^-dependent class III Sir2 and the zinc-dependent class II Hdal (15). *H. volcanii* encodes a total of 29 proteins containing acetyltransferase domains, including Pat1, Pat2, and Elp3, though their specific acetylation targets remain unidentified (16). *H. volcanii* Pat1 and Pat2 are of interest due to their differential abundance under oxidative stress. Upon *H. volcanii* challenge with hypochlorite, Pat1 levels decrease 3.1-fold, while Pat2 levels increase 1.8-fold, suggesting a potential correlation between KAc and oxidative stress response (14). In addition, the KAc signal is increased upon exposure to oxidants (hypochlorite) (12). While deletion of the *pat1* and *pat2* genes decreases certain KAc modifications, lysine acetylation still occurs regardless of this double mutation (12). Moreover, KAc signals are still increased in abundance by treatment with hypochlorite (12). These results indicate that additional acetyltransferases or non-enzymatic mechanisms contribute to KAc modifications.

While *Hv*Elp3 is of the GNAT family, it is also related to and annotated as a tRNA carboxymethyluridine synthase (17). In eukaryotic systems, Elp3 is the catalytic subunit of the Elongator (Elp) complex, which catalyzes uridine modifications at the wobble position (18). This subunit contains two putative domains: radical S-adenosylmethionine (SAM) at the N-terminus and the histone acetyltransferase (HAT or GNAT) domain at the C-terminus (19, 20). A study in the archaea *Methanocaldococcus infernus* aimed to perform an *in vitro* reconstitution of radiolabeled acetyl-CoA and synthetic tRNA. The results suggest the C-terminal GNAT domain of Elp3 is utilized for adding a carboxymethyl (cm^5^) group to uridine at the wobble position, reproposing Elp3 is not a lysine acetyltransferase but instead is involved with wobble uridine tRNA modification (21).

Due to the close evolutionary relationship of archaea with eukaryotes, as well as the genome sequenced, *H. volcanii* has emerged as a model organism with significant advancements widely used in genetic, molecular, and biochemical research to provide valuable insights into cellular mechanisms of survival under extreme environmental conditions (22). Insights gained from studying *H. volcanii* have not only expanded our understanding of archaeal biology but will also provide knowledge to the mechanisms of post-translational modifications in prokaryotes. Previous efforts aimed to generate a Δ*pat2*Δ*elp3* double mutant suggested that these genes are involved in synthetic lethal interaction, in which their products share the same targets, and deemed essential for *H. volcanii* viability (15). Here, we provide genetic and phenotypic evidence in *H. volcanii* that *elp3* can be deleted in combination with either *pat1* or *pat2*, thus supporting a conclusion that a Δ*pat2*Δ*elp3* double mutation is not lethal.

## MATERIALS AND METHODS

### Materials

Biochemicals were purchased from Fisher Scientific (Atlanta, GA, USA), Bio-Rad (Hercules, CA, USA), and Sigma-Aldrich (St. Louis, MO, USA). Oligonucleotides and DNA Sanger sequencing services were purchased from Eurofins Genomics (Louisville, KY, USA). Phusion High-Fidelity DNA Polymerase, restriction enzymes, DpnI, kinase, ligase and DpnI (KLD) enzyme mix, and 2× Quick Ligase were purchased from New England Biolabs (NEB) (Ipswich, MA, USA). DNA fragments were isolated using NEB Monarch PCR and DNA Clean-up Kit or DNA Gel Extraction Kit (Ipswich, MA, USA).

### Plasmid construction and generation of deletion strains

Strains, primers, and plasmids used in this study are listed in Tables 1–3. Genomic DNA for plasmid strain construction was extracted from *H. volcanii* by a DNA spooling method (23). The pTA131-based pre-deletion plasmids were generated by restriction enzyme digestion and ligation of a DNA fragment containing the flanking regions 500 bp 5′ (upstream) and 3′ (downstream) of the gene of interest. The knock-out plasmids were constructed by inverse PCR and gel extracted, then treated with DpnI and PCR clean-up before adding KLD enzyme mix according to the manufacturer’s instruction (New England Biolabs, Ipswich, MA). Plasmids were transformed into *Escherichia coli* Top10, *E. coli* GM2163, and *H. volcanii* H1207. Deletion plasmids to generate Δ*elp3* mutations were transformed into *H. volcanii* H1207, KT08, and KT09 to generate KT10, KT17, and KT18, respectively.

**TABLE 1:** List of plasmids used for *H. volcanii* gene deletions in this study

Plasmid	Description	Source
pTA131	Amp^r^; pBluescript II containing P_*fdx*_-*pyrE2*	(24)
pJAM4009	Amp^r^; pTA131 carrying *pat1* and ~500 bp flanking sequence (pre-KO plasmid)	This study
pJAM4013	Amp^r^; pJAM4009 with Δ*pat1* (deletion plasmid)	This study
pJAM4010	Amp^r^; pTA131 carrying *pat2* and ~500 bp flanking sequence (pre-KO plasmid)	This study
pJAM4014	Amp^r^; pJAM4010 with Δ*pat2* (deletion plasmid)	This study
pJAM4463	Amp^r^; pTA131 carrying *elp3* and ~500 bp flanking sequence (pre-KO plasmid)	This study
pJAM4464	Amp^r^; pJAM4463 with Δ*elp3* (deletion plasmid)	This study

**TABLE 2:** List of strains generated and used in this study

Strain	Description	Source
DS70	Wild-type isolate DS2 cured of plasmid pHV2	(25)
H26	DS70 Δ*pyrE2*	(24)
H1207	H26 Δ*pyrE2 pitA*_Nph_ Δ*mrr*	(26)
KT08	H1207 Δ*pat1*	This study
KT09	H1207 Δ*pat2*	This study
KT10	H1207 Δ*elp3*	This study
KT11	H1207 Δ*pat1*Δ*pat2*	This study
KT17	H1207 Δ*pat1*Δ*elp3*	This study
KT18	H1207 Δ*pat2*Δ*elp3*	This study

**TABLE 3:** List of primers used in this study compared to Altman-Price and Mevarech (15)

No.	Primer	Sequence	Source
1	Pat1 knock-in (BamHI)	ATCGGATCCGCGTTGCCGAGGTAGAAGAACGTC	This study
2	Pat1 knock-in (HindIII)	TTTAAGCTTCGAACGCGGACTGAGCGCCTCGGA	This study
3	Pat1 inverse knock-out F	GCGGACACGAAGATGCGTCTCGACCTGAC	This study
4	Pat1 inverse knock-out R	CGGTCAACGTCCGTCTCTCCCATACG	This study
5	Pat1 external F	TTCGAGAGAAACGATAGCCGAGCGCGGCCG	This study
6	Pat1 external R	GTTTTAGCCGGCGACCCGCTACGCTCGACC	This study
7	Pat1 internal R	CGTGGCCGACGATACCTCGGTCGGCGAC	This study
8	Pat1 internal F	CCGGCCGAACGGCGCGAGTGGTTCCGAC	This study
9	Pat2 knock-in (BamHI)	TTTGGATCCGGACTCGTCTGTCATACCGCGGGC	This study
10	Pat2 knock-in (HindIII)	TTTAAGCTTCGCGCCCGCTCTCTATCGACCTCG	This study
11	Pat2 inverse knock-out F	CCGGACGACGAGGGCGGCTGAGGGCG	This study
12	Pat2 inverse knock-out R	CGTACTAGGGTGACGGTCACGTGGGAGTTCAACAGGACG	This study
13	Pat2 external F	CCGGACCTTGTCGCGGAACGCAGACCGGG	This study
14	Pat2 external R	CCGCTCTGCGAGGTCGACGGCGACGCTG	This study
15	Pat2 internal F	CGTCGAGATGTACGACGCGTTCGACCCCT	This study
16	Pat2 internal R	CGGTTCCAGCGCTCGACCGTGAGCCAC	This study
17	Elp3 knock-in (Xhol)	CCGCTCGAGGTCGCGTTGGAAGCCTACTA	This study
18	Elp3 knock-in (BamHI)	CGCGGATCCCGCGTACGAGTCCAGTTTCT	This study
19	Elp3 inverse knock-out F	CCGACGAGCGCTCTCCTGCCGATTC	This study
20	Elp3 inverse knock-out R	GGCCACACCTCCCGTTCAGCGACGTGG	This study
21	Elp3 external F	CTTCTCCTCGGTGCCGGTTCGACCGC	This study
22	Elp3 external R	CGAACGGTCGGGAGAACATCAGCAC	This study
23	Elp3 internal R	CCACGACTACCAGGAGTGGTTCG	This study
24	Elp3 internal F	GGGCTGACCGGGCATCATGTG	This study
25	5′up Pat1(KI) (XbaI)	TCTAGATTCGAGAGAAACGATAGCCGA	(15)
26	3′up Pat1 (KO) (BamHI)	GGATCCGCTTCACATGTGAGGTGACAG	(15)
27	5′down Pat1 (KO) (SphI)	GCATGCCCGGACTCCCGGCGAACCTCA	(15)
28	3′down Pat1 (KI) (HindIII)	AAGCTTCGTTCGACTCGCCGCCGGCGA	(15)
29	5′up Pat2 (KI) (XbaI)	TCTAGACGACGCGCGGTCCCCCGCTG	(15)
30	3′up Pat2 (KO) (BamHI)	GGATCCTCATCGTACTAGGGTGACGG	(15)
31	5′down Pat2 (KO) (SphI)	GCATGCGGGCGGCGGCGAGCCGCGAC	(15)
32	3′down Pat2 (KI) (HindIII)	AAGCTTAGTACGACGCCGCGGTCCAC	(15)
33	5′up Elp3 (KI) (XbaI)	TCTAGATCAGGACAAGCGCGAACTCA	(15)
34	3′up Elp3 (KO) (BamHI)	GGATCCACCTCCCGTTCAGCGACGT	(15)
35	5′down Elp3 (KO) (SphI)	GCATGCCCGACGAGCGCTCTCCTGC	(15)
36	3′down Elp3 (KI) (HindIII)	AAGCTTAGACGATGCCCTGCTGGTG	(15)

The *H. volcanii* deletion mutants were generated by the *pyrE2* pop-in/pop-out homologous recombination method as described (24) with the following modifications. Transformants were plated on Hv-Ca^+^ during the pop-in stage, and successful plasmid integration was determined via PCR screening. Positive colonies were grown in 5 mL ATCC974 supplemented with 50 µg/mL 5-fluoroorotic acid (5-FOA) [diluted throughout this study from a 50 mg/mL 5-FOA stock dissolved indimethylsulfoxide (DMSO)] in 13 × 100 mm culture tubes and grown in the dark at 42°C with orbital shaking at 200 rpm for 3–4 days. Dilution series (10^−3^ to 10^−6^) of this culture was plated on ATCC974 supplemented with 50 µg/mL 5-FOA and 1.5% (wt/vol) agar. Genome deletion was determined by PCR screening. Colonies displaying deletion were streaked for isolation four additional times on ATCC974 supplemented with 50 µg/mL 5-FOA. This technique was employed to generate single and double deletions of *pat1* (HVO_1756), *pat2* (HVO_1821), and *elp3* (HVO_2888). Final deletion was monitored by PCR with check primers containing 5′ and 3′ flanking regions 700 bp 5′ (upstream) and 3′ (downstream) of the gene of interest, as well as 300–400 bp internally.

### DNA extraction for genome sequencing

Strains were inoculated from 20% (vol/vol) glycerol stocks (−80°C) onto ATCC974 medium supplemented with 1.5% (wt/vol) agar and grown at 42°C for 5 days. The glycerol stocks were generated by performing a 1:4 dilution of stationary phase cultures with a solution of 80 mL 100% glycerol supplemented with 20 mL of 30% salt H_2_O and 0.2 mL of 0.5 M CaCl_2_. From the plates, a single colony was cultured with 5 mL ATCC974 medium in 13 × 100 mm culture tubes and grown until OD_600_ 0.6. To a 2 mL Eppendorf tube, 2 mL of cell culture was pelleted and frozen at 80°C until further use. Genomic DNA was extracted by ThermoScientific GeneJET Genomic DNA Purification Kit (Atlanta, GA, USA) according to manufacturer’s instructions and eluted in H_2_O.

### Whole-genome sequencing

Sequencing libraries were prepared using the tagmentation-based and PCR-based Illumina DNA Prep kit, incorporating custom 10 bp unique dual indices from Integrated DNA Technologies (IDT, Coralville, IA, USA) with a target size of 280 bp. Illumina whole-genome sequencing (200 Mbp) was performed. The paired-end sequencing data generated by Illumina were used for variant calling against the reference genome *H. volcanii* DS2 (NCBI accession NC_013967.1 [SeqCenter, Pittsburgh, PA, USA]). Sequencing was performed with an Illumina NovaSeq X Plus platform using multiplexed shared-flow-cell runs, generating paired-end reads of 2 × 151 bp. For quality control, demultiplexing, and adapter trimming, bcl-convert (v.4.2.4) was used. Variant calling was carried out using BreSeq (version 0.38.1) with the default settings. No parameter adjustments were made during the variant calling process (SeqCenter, Pittsburgh, PA).

### PCR deletion screening

PCR was performed with Phusion High-Fidelity DNA polymerase according to manufacturer’s instructions with 10 s extension phases for 25× cycles for all internal primers. The external primers were performed with 1.5 min for *elp3* and 30 sec for *pat1* and *pat2* extension phase for 25 × cycles (T100 ThermoCycler, Bio-Rad). PCR products were compared to GeneRuler 1 kb plus DNA ladder (cat# SM1331, Thermo Fisher, Waltham, MA, USA). DNA fragments were separated by 0.8% (wt/vol) agarose gel supplemented with 0.0025% (vol/vol) ethidium bromide electrophoresis (90 V and 30 min) in 1× TAE buffer (0.001% [vol/vol] ethidium bromide, 40 mM Tris, 20 mM acetic acid, 1 mM EDTA, pH 8.0). DNA agarose gels were imaged using the iBright FL 1000 imaging system (Thermo Fisher) on the nucleotide setting.

### Growth curve analysis

Strains were inoculated from 20% (vol/vol) glycerol stocks (−80°C) onto ATCC974 rich medium supplemented with 1.5% (wt/vol) agar. Plates were incubated at 42°C for 5 days. Single colonies were cultured with 5 mL ATCC974 medium in rotating culture tubes (13 × 100 mm). Cells were grown to log phase (OD_600_ 0.6–0.8) and sub-cultured to OD_600_ 0.02 with 5 mL ATCC974 medium once or HY-rich medium twice and grown to log phase. Cells were sub-cultured again to OD_600_ 0.02 with 1 mL respective medium in 1.5 mL Eppendorf tubes and briefly (5–10 min) incubated with rotation at 42°C prior to aliquoting. In a 96-well CellPro cell culture plate (Alkali Scientific, FL), 150 µL of subculture was aliquoted into six replicate wells. Using the BioTek Epoch 2 microplate reader and Gen5 software (Agilent, Santa Clara, CA), cell growth was measured as follows: OD_600_ was measured every 15 min for 99 h, with aeration (double orbital continuous shaking) and temperature setpoint 42°C. No inoculum controls were included to assess potential background signals from the medium alone. ATCC974 medium, pH 6.8, was composed per liter of 125 g NaCl, 50 g MgCl_2_ ∙6H_2_O, 5 g K_2_SO_4_, 0.134 g CaCl_2_ ∙2H_2_O, 5 g tryptone, and 5 g yeast extract. HY-rich medium was composed per liter of 150 g NaCl, 36.9 g MgSO_4_ · 7H_2_O, 5 mL of 1 M KCl solution, 1.8 mL of 75 mg/L MnCl_2_ solution, 5 g yeast extract, 50 mM Tris-HCl (pH 7.2), and once cooled 5 mL of 10% (wt/vol) CaCl_2_ (15).

### Structural modeling of *Hv*Elp3

The 3D structural model of *Hv*Elp3 was generated using two independent computational prediction platforms: AlphaFold Server 3 (27) and Phyre2 (28). An AlphaFold-predicted *Hv*Elp3 structure with a high degree of confidence, as indicated by a predicted template modeling score of 0.89, was used for comparison to Elp3 proteins with experimentally determined 3D structures. Phyre2 was used for homology-based structural modeling (confidence in the model: 100.0%). All structural visualizations and analyses were conducted using ChimeraX (version 1.9) (29). To assess electrostatic surface properties, the predicted structure was visualized using Coulombic surface coloring in ChimeraX, where negatively charged regions are represented in red, positively charged regions in blue, and neutral regions in white. Chimera X matchmaker option was used to determine the root mean square deviation (RMSD) values.

## RESULTS

### Structural conservation of Elp3 homologs across diverse organisms

**Fig 1:**
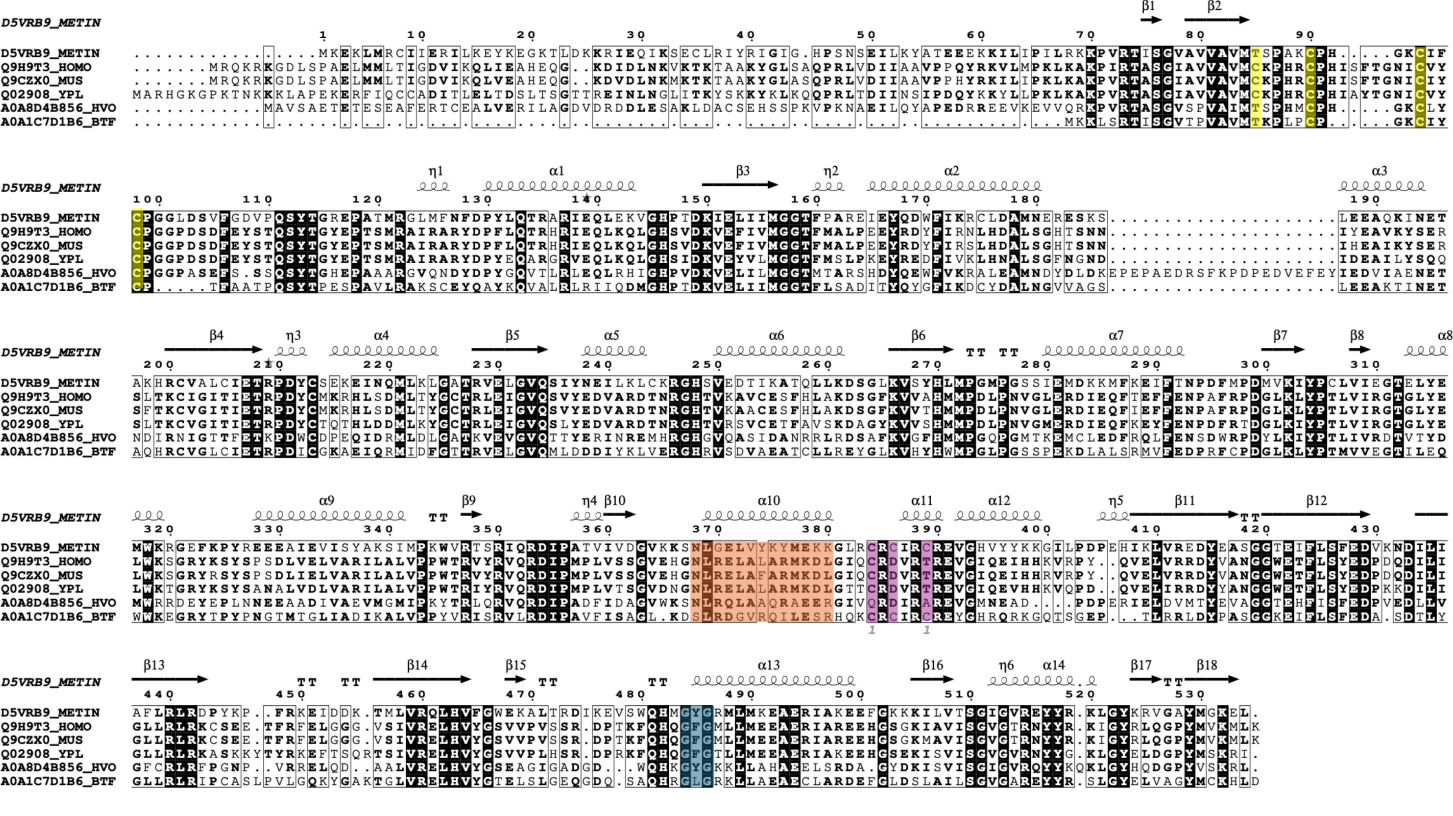
Sequence alignment of *Hv*Elp3 with representative Elp3 enzymes from all domains of life. Abbreviations: *H. volcanii* (HVO), *Homo sapiens* (HOMO), *Methanocaldococcus infernus* (METIN)*, Saccharomyces cerevisiae S288C* (YPL)*, Mus musculus* (MUS)*,* and *Dehalococcoides mccartyi* BTF08 (BTF). *Hv*Elp3 contains a 22 amino acid insertion at positions 188–209. Cysteine residues coordinating iron-sulfur binding are highlighted in yellow. *D. mccartyi* Elp3 C27 and C30 coordinate the Fe-S, while the conserved C23 is not involved in coordination (34). The acetyl-CoA G-X-G motif (in blue) corresponds to the “acetyl-CoA loop” of *H. sapiens* Elp3, which spans residues 477–497 (31) and the “acetyl-CoA blocking loop” of *M. infernus* Elp3, which includes residues 466–486 (19). The zinc-binding residues C310, C312, and C315 of *D. mccartyi* Elp3 (34), while not conserved in *Hv*Elp3, are conserved within *M. infernus* Elp3 (as highlighted in purple). Although *S. cerevisiae* Elp3 does not contain a zinc-binding motif, this protein has a conserved region from 391 to 403 that points toward the direction of the central active site cavity (orange) (36). Multialign (37) was used to generate the sequence alignment and imported to ESPript 3.0 (38). *M. infernus* PDBe ID 6ia8 was used as the input file for the secondary structure depiction.

This study first aimed to compare the conserved regions and putative active sites of *Hv*Elp3 to provide further insight into its potential biological role. Structural homology modeling using Phyre2 (28) and AlphaFold (27) indicates that *Hv*Elp3 shares similarity with Elp3 proteins across all domains of life ( Table 4). Sequence alignment of structurally similar homologs reveals conserved regions among the six Elp3 proteins analyzed (Fig. 1). In *Saccharomyces cerevisiae*, the Elp3 features a C-terminallysine acetyltransferase (KAT) domain and a cysteine-rich iron-sulfur (Fe-S) cluster motif within the N-terminal SAM-binding domain (30). The four cysteines that coordinate the Fe-S cluster are also conserved in *Homo sapiens* (31) and *Mus musculus* (32)*,* where the cluster is proposed to facilitate SAM binding (33). In *M. infernus*, three cysteines coordinate a 2Fe-2S cluster that is similarly located within the N-terminal SAM-binding domain, and the presence of both SAM and KAT domains enables Elp3 to catalyze acetyl-CoA hydrolysis (19). The Elp3 homolog of *Dehalococcoides mccartyi*, which uses only two cysteine residues to coordinate an Fe-S cluster (34), has a five-amino-acid deletion after the CP motif that is not observed in the other Elp3 homologs analyzed. In *D. mccartyi*, the two Elp3 domains are linked by a zinc-binding motif, which contributes to the formation of the active site and the tRNA-binding pocket (34). *Hv*Elp3 has structural features consistent with coordinating a Fe-S cluster via the conserved CP-X_3_-C-X_2_-CP motif located in its N-terminal SAM binding domain. *Hv*Elp3 also contains the conserved acetyl-CoA G-X-G-binding motif spanning residues 502–504, which is found across all species examined. However, the cysteine residues of the *D. mccartyi* Elp3 involved in zinc binding (C310, C312, and C315), while conserved in the *M. infernus* Elp3, are not conserved in *Hv*Elp3 or the eukaryotic Elp3s. When comparing the *Hv*Elp3 residues (394 to 406) predicted to be oriented toward the central active site cavity, there is 46% identity to the eukaryotic Elp3s.

**TABLE 4:** Comparison of 3D structures of *Hv*Elp3 models predicted by Phyre2 and AlphaFold to CryoEM and X-ray crystallographic structures of Elp3 homologs^*c*^

Organism	Protein	PDBe; UniProt ID	AA	Seq. Id.	Prob.	E-value	RMSD (atom pairs)—Phyre2^*a*^	RMSD (atom pairs)—AlphaFold^*a*^
*Haloferax volcanii*	Elp3	– A0A8D4B856	552	–	–	–	1.177 Å (394);5.699 Å (520)	
*Homo sapiens*	Elp3^*b*^	8ptx; Q9H9T3	547	–	–	–	0.209 Å (475); 1.721 Å (516)	1.148 Å (396); 5.853 Å (523)
*Methanocaldococcus infernus*	Elp3	6ia8; D5VRB9	534	41.6	1.0	1.79e^−46^	1.152 Å (325); 2.742 Å (417)	0.690 Å (378); 3.824 Å (417)
*Saccharomyces cerevisiae S288C*	Elp3^*b*^	6qk7; Q02908	557	40.2	1.0	5.62e^−46^	0.779 Å (410); 1.412 Å (433)	0.986 Å (387); 2.991 Å (433)
*Mus musculus*	Elp3^*b*^	8avg; Q9CZX0	547	39.1	1.0	4.95e^−42^	0.788 Å (400); 1.446 Å (426)	1.075 Å (376); 2.737 Å (426)
*Dehalococcoides mccartyi BTF08*	Elp3	5l7l; A0A1C7D1B6	459	35.9	1.0	3.77e^−36^	1.130 Å (267); 3.328 Å (389)	0.770 Å (326); 2.975 Å (389)

^*a*^ HvElp3 Phyre2 (28) and AlphaFold (27) generated models compared to each other and to other homologs as indicated. RMSD values based on pruned and total atom pairs calculated using ChimeraX (29). Sequence identity (Seq. Id.), probability (Prob.), and E-value metrics determined by comparison of *Hv*Elp3 AlphaFold model (27) to Elp3 homologs using Foldseek (35).

^*b*^ Elp3 of eukaryotes represents the Elp3 subunit of the Elp123 complex.

^*c*^ –, not applicable; PDBe, PDB entry; UniProt ID, accession number; AA, amino acid length.

### Single and double mutations of *pat1*, *pat2,* and *elp3* in *H. volcanii*

**Fig 2:**
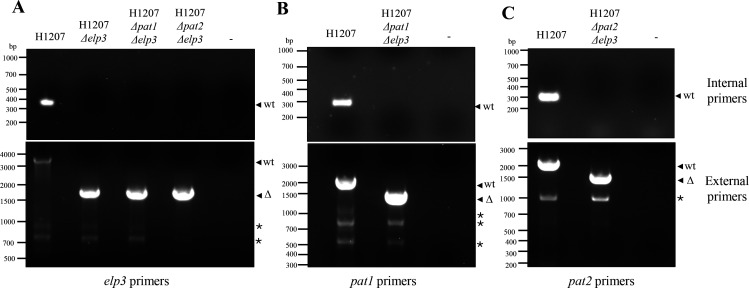
*H. volcanii* strains with single and combined deletions of *elp3* with *pat1* or *pat2* are viable. Parent (H1207) and Δ*elp3*, Δ*pat1*Δ*elp3*, and Δ*pat2Δelp3* mutant strains demonstrated by PCR screen. Cells were inoculated from 20% (v/v) glycerol stocks (−80°C) onto ATCC974 medium supplemented with 1.5% (w/v) agar. A single isolated colony was transferred to a 0.5 mL microcentrifuge tube with 50 µL of sterile H_2_O, heated to 95°C for 10 min, and cooled at 4°C for 5 min. The cell material was used for PCR, and sterile H_2_O was used for the negative control (−). Deletion of the *elp3* (**A**), *pat1* (**B**), and *pat2* (**C**) genes was monitored by PCR with internal primers (upper panels) and external primers (lower panels), as indicated. The expected sizes for the internal primers were as follows: *elp3* at 400 bp, *pat1* at 302 bp, and *pat2* at 312 bp. The internal primer annealing temperatures were as follows: *elp3* 68°C, *pat1* 74°C, and *pat2* 74°C. The expected sizes for the external primers were as follows: *elp3* parent expected 3,401 bp vs deletion 1,748 bp, *pat1* parent expected 2,023 bp vs deletion 1,506 bp, *pat2* parent expected 2,188 bp vs deletion 1,654 bp. The external primer annealing temperatures were as follows: *elp3* 67°C, *pat1* 74°C, and *pat2* 74°C.

The essentiality of the *H. volcanii* HAT gene homologs was re-evaluated using a pop-in/pop-out method (23) with the strategy to generate markerless deletions of the genes of interest. The plasmids used to target *pat1* (pJAM4013), *pat2* (pJAM4014), and *elp3* (pJAM4464) for deletion (Table 1) were transformed into the *H. volcanii* parent strain H1207 to generate single-gene deletions. Subsequently, the *elp3* deletion plasmid was introduced into the single mutant strains KT08 (H1207 Δ*pat1*) and KT09 (H1207 Δ*pat2*) to construct double mutants. The final strains generated are summarized in Table 2. Selection for the *pyrE2* marker was conducted on Hv-Ca^+^ (uracil minus) minimal medium, followed by counterselection on ATCC974 rich medium supplemented with 5-FOA. Each strain underwent counterselection through four consecutive isolation streaks with PCR screening at each step to monitor successful deletion of the target gene. To further validate the deletions, the strains were recovered from 20% (vol/vol) glycerol stocks stored at −80°C and plated onto ATCC974 rich medium to confirm the absence of the wild-type gene. Successful deletion was monitored by PCR analysis using external and internal gene-specific primers (Fig. 2). The PCR results demonstrated the generation of both single and double mutants of Δ*pat1*, Δ*pat2*, and Δ*elp3*, with no PCR-detectable wild-type signal present.

### Whole-genome sequencing confirms deletion of *pat2* and *elp3*

To further support the PCR-based screening and verify the successful deletion of the *pat2* and *elp3* genes in a single strain, whole-genome sequencing was performed. Genomic DNA from H1207 and KT18 (H1207 Δ*pat2*Δ*elp3*) was sequenced and analyzed through variant calling against the reference genome (*H. volcanii* DS2; NCBI accession NC_013967.1) (Data Set S1). The DNA sequence analysis confirmed the successful deletion of 534 bp of *pat2* (GNAT family N-acetyltransferase HVO_RS13445) and 1,653 bp of *elp3* (tRNA [uridine 34] 5-carboxymethylaminomethyl modification radical SAM/GNAT enzyme HVO_RS18670) in the KT18 mutant compared to the H1207 parent. According to NCBI (24), *pat2* is 555 bp and *elp3* is 1,659 bp (39). Additional mutations unique to KT18, relative to H1207, were identified. These additional mutations were insertions at genomic positions 1,697,788 +C, 1,697,869 +A, and 1,697,871 +TGCTCAG that were associated with the ISH3-like element ISHvo20 family transposase pseudogene (*hvo_RS20195*). These mutations within a pseudogene are unlikely to confer suppressor effects during Δ*pat2*Δ*elp3* mutagenesis and are more likely the result of random genomic DNA drift (27). Sequencing coverage for H1207 and H1207 Δ*pat2*Δ*elp3* was 374× and 404×, respectively. Coverage was calculated as the read length (paired-end 150 bp reads) multiplied by the total number of reads for the sample, divided by the genome size (~2.8 Mbp).

### Phenotypic analysis reveals Δ*elp3* single and double mutants with Δ*pat1* or Δ*pat2* are viable but have growth defects

**Fig 3:**
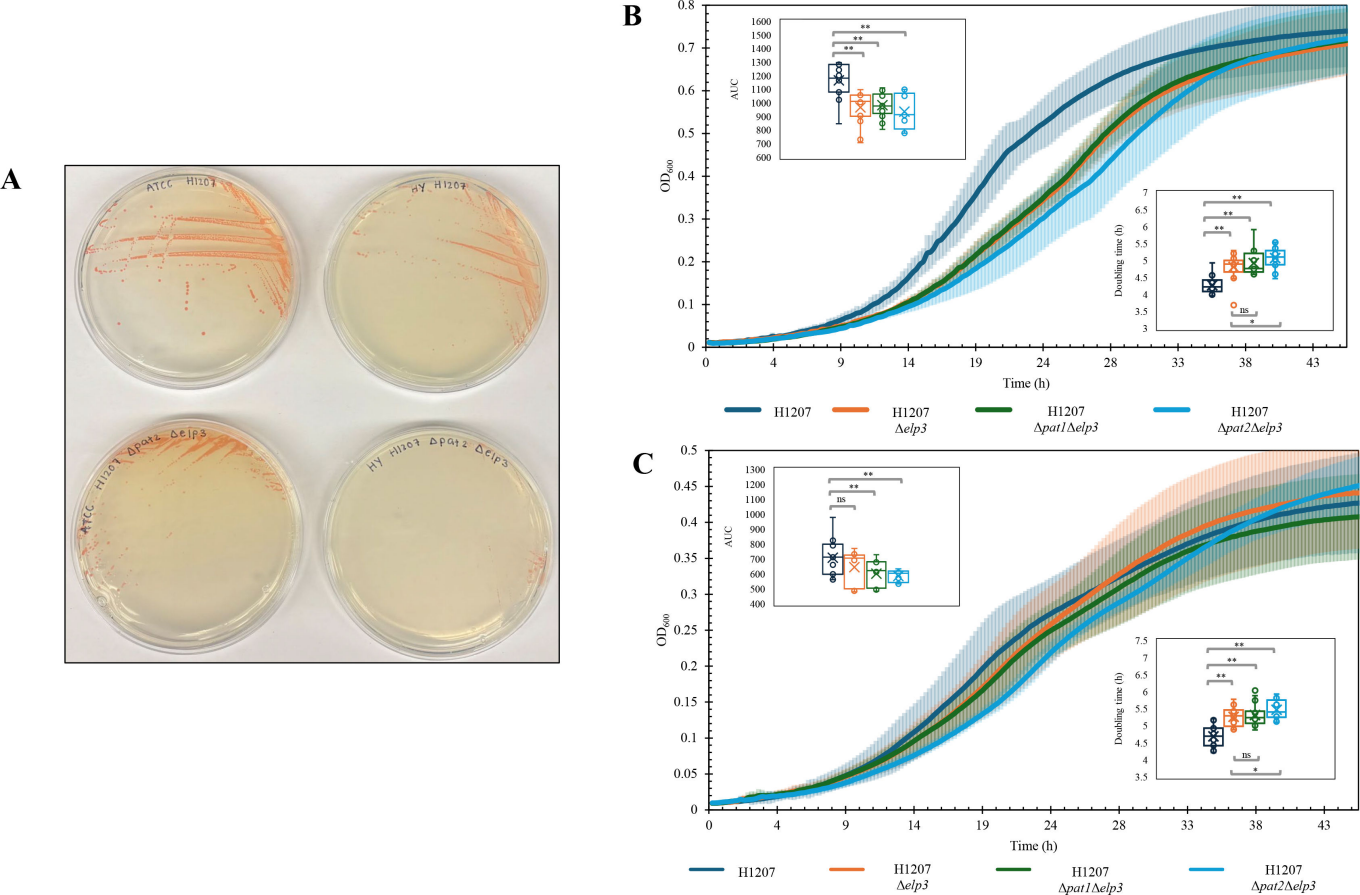
Strains with single and combined deletions of *elp3* with *pat1* or *pat2* are viable but exhibit reduced growth compared to the parent strain (H1207). (**A**) *H. volcanii* strains grown on HY-rich medium display growth defects. Parent (H1207) and Δ*pat2*Δ*elp3* mutant strain cells were inoculated from 20% (vol/vol) glycerol stocks (−80°C) onto ATCC974 and HY-rich medium supplemented with 1.5% (wt/vol) agar. Plates were grown at 42°C for 8 days. *H. volcanii* strains were inoculated from 20% (vol/vol) glycerol stocks (−80°C) onto ATCC974-rich medium plates and incubated for 5 days at 42°C. Isolated colonies were transferred to 5 mL of ATCC974 and grown to log phase at 42°C in rotating culture tubes (13 × 100 mm). (**B**) Cells were sub-cultured into 5 mL of fresh ATCC974 media at OD_600_ 0.02 and incubated until log phase (OD_600_ 0.6–0.8) at 42°C. For growth monitoring, cells were further sub-cultured into ATCC974 medium in microtiter plates (96-well, OD_600_ of 0.02), and growth was measured at OD_600_ at 15 min intervals using an Epoch 2 Biotech microtiter plate reader at 42°C with aeration (double orbital continuous shaking). The growth curve represents three technical replicates and six biological replicates each. No-inoculum control was used as a blank. The experiment was reproducible. A Student’s *t*-test was used to determine the statistical significance (*P*-value <0.005, **; *P*-value <0.05, *; *P*-value >0.05, ns) of the AUC average values calculated over the 46.5 h time course for the parent (H1207, AUC 1165 ± 118) compared to Δ*elp3* (*P*-value 5.54 × 10^−6^, AUC 966 ± 113), Δ*pat1*Δ*elp3* (*P*-value 6.20 × 10^−6^, AUC 983 ± 88), and Δ*pat2*Δ*elp3* (*P*-value 2.25 × 10^−6^, AUC 936 ± 124) mutant strains. Student’s *t*-test was used to determine the statistical significance (*P*-value <0.005, **; *P*-value <0.05, *; *P*-value >0.05, ns) of doubling time average values calculated over the 30 h time course for the parent (H1207, 4.27 ± 0.23 h) compared to Δ*elp3* (*P*-value 3.91 × 10^−6^, 4.81 ± 0.36 h), Δ*pat1*Δ*elp3* (*P*-value 1.65 × 10^−7^, 4.92 ± 0.36 h), and Δ*pat2*Δ*elp3* (*P*-value 3.01 × 10^−10^, 5.07 ± 0.30 h) mutant strains. The *t*-test was also used to determine the significance of Δ*pat2*Δ*elp3* compared to Δ*elp3* (*P*-value 0.01) and Δ*pat1*Δ*elp3* (*P*-value 0.11). (**C**) Cells were sub-cultured into 5 mL of HY-rich medium (15) at OD_600_ 0.02 and incubated until log phase at 42°C. Cells were sub-cultured again, ensuring removal of ATCC974 media, into 5 mL of HY-rich medium at OD_600_ 0.02 and incubated until log phase at 42°C. For growth monitoring, cells were further sub-cultured into HY-rich medium in microtiter plates (96-well, OD_600_ of 0.02) and growth was measured at OD_600_ at 15 min intervals using an Epoch 2 Biotech microtiter plate reader at 42°C with aeration (double orbital continuous shaking). The growth curve represents three technical replicates and six biological replicates each. No-inoculum control was used as a blank. The experiment was reproducible. Student’s *t*-test was used to determine the statistical significance (*P*-value <0.005, **; *P*-value <0.05, *; *P*-value >0.05, ns) of the AUC average values calculated over the 46.5 h time course for the parent (H1207, AUC 708.96 ± 112.27) compared to Δ*elp3* (*P*-value 0.051, AUC 646.08 ± 111.90), Δ*pat1*Δ*elp3* (*P*-value 1.58 × 10^−3^, AUC 608.83 ± 86.84), and Δ*pat2*Δ*elp3* (*P*-value 1.15 × 10^−4^, AUC 592.12 ± 39.77) mutant strains. Student’s *t*-test was used to determine the statistical significance (*P*-value <0.005, **; *P*-value <0.05, *; *P*-value >0.05, ns) of doubling time average values calculated over the 30 h time course for the parent (H1207, 4.69 ± 0.29 h) compared to Δ*elp3* (*P*-value 4.12 × 10^−7^, 5.27 ± 0.28 h), Δ*pat1*Δ*elp3* (*P*-value 4.70 × 10^−7^, 5.32 ± 0.31 h), and Δ*pat2*Δ*elp3* (*P*-value 3.37 × 10^−8^, 5.46 ± 0.26 h) mutant strains. The *t*-test was also used to determine the significance of Δ*pat2*Δ*elp3* compared to Δ*elp3* (*P*-value 0.03) and Δ*pat1*Δ*elp3* (*P*-value 0.09). Dark blue, H1207; orange, H1207 Δ*elp3*; green, H1207 Δ*pat1*Δ*elp3*; light blue, H1207 Δ*pat2*Δ*elp3*.

While deletion of *elp3*, either alone or in combination with the Δ*pat1* or Δ*pat2* mutations, was not lethal, it did reduce growth compared to the parent (H1207) in ATCC974 and HY-rich media. Visible differences in growth were apparent on solid media: the Δ*pat2*Δ*elp3* mutant formed fewer visible colonies than the parent strain on HY medium, whereas on ATCC medium, both strains exhibited similar colony size, density, and color. The Δ*pat2*Δ*elp3* mutant strain exhibited no colony formation on HY-rich medium by day 6, whereas growth was observed on ATCC974. However, by day 8, slight growth is apparent on HY-rich medium (Fig. 3A). When OD_600_ was used to monitor growth in liquid culture (Fig. 3B and C), all three mutant strains (Δ*elp3*, Δ*pat1*Δ*elp3,* and Δ*pat2*Δ*elp3*) displayed reduced growth rates, increased doubling times, and lower area under the curve (AUC) values when compared to the parent (H1207) in the ATCC974 and HY media (Table 5). Additionally, the Δ*pat2*Δ*elp3* mutant grew slower than Δ*elp3* alone in both types of media. In support of the use of ATCC974 for the final stages of generating the Δ*pat2*Δ*elp3* double mutation, the *H. volcanii* strains generally reached a twofold higher optical density at 600 nm when grown on ATCC974 compared to HY media. Overall, these findings suggest that *elp3* and *pat2* influence cellular fitness but are not a synthetic lethal gene pair.

**TABLE 5:** Area under the curve (AUC), growth rates, and doubling times of parent (H1207) and mutant strains cultivated in ATCC974 and HY media

Strain	ATCC974 medium			HY medium		
	AUC	Growth rate (h^−1^)	Doubling time (h)	AUC	Growth rate (h^−1^)	Doubling time (h)
H1207	1,165 ± 118	0.162 ± 0.01	4.27 ± 0.23	708.96 ± 112.27	0.148 ± 0.01	4.69 ± 0.29
Δ*elp3*	966 ± 113	0.144 ± 0.01	4.81 ± 0.36	646.08 ± 111.90	0.132 ± 0.01	5.27 ± 0.28
Δ*pat1*Δ*elp3*	983 ± 88	0.141 ± 0.01	4.92 ± 0.36	608.83 ± 86.84	0.131 ± 0.01	5.32 ± 0.31
Δ*pat2*Δ*elp3*	936 ± 124	0.137 ± 0.01	5.07 ± 0.30	592.12 ± 39.77	0.127 ± 0.01	5.46 ± 0.26

## DISCUSSION

A previous study (15) aimed to generate single and double mutants of the three lysine acetyltransferase homologs *pat1*, *pat2*, and *elp3* of *H. volcanii*. In that study (15), the Δ*pat2*Δ*elp3* mutant was not successfully generated, deeming that the deletion of both genes was considered synthetically lethal due to the potential of their products sharing mutual targets. In this study, we show that *H. volcanii* strains with *elp3* mutations combined with *pat1* or *pat2* deletions remain viable. Whole-genome sequencing further validates the successful double deletion of *pat2* and *elp3* at the genomic level and provides a comprehensive profile of the engineered strains, supporting their suitability for further exploration of lysine acetyltransferase function. In this study, the *pat2* deletion removed four nucleotides at the 3′-end of *uspA* (*hvo_1820*), while simultaneously keeping 21 nucleotides of the 3′-end of *pat2*, generating a *uspA* frame shift that encoded UspA with a C-terminal 85 additional amino acid extension. According to 3D modeling, the additional amino acids do not impact UspA folding (Fig. S1). The potential impact of these terminal residues on UspA functionality and Pat2/Elp3 deletion feasibility is currently unknown.

**Fig 4:**
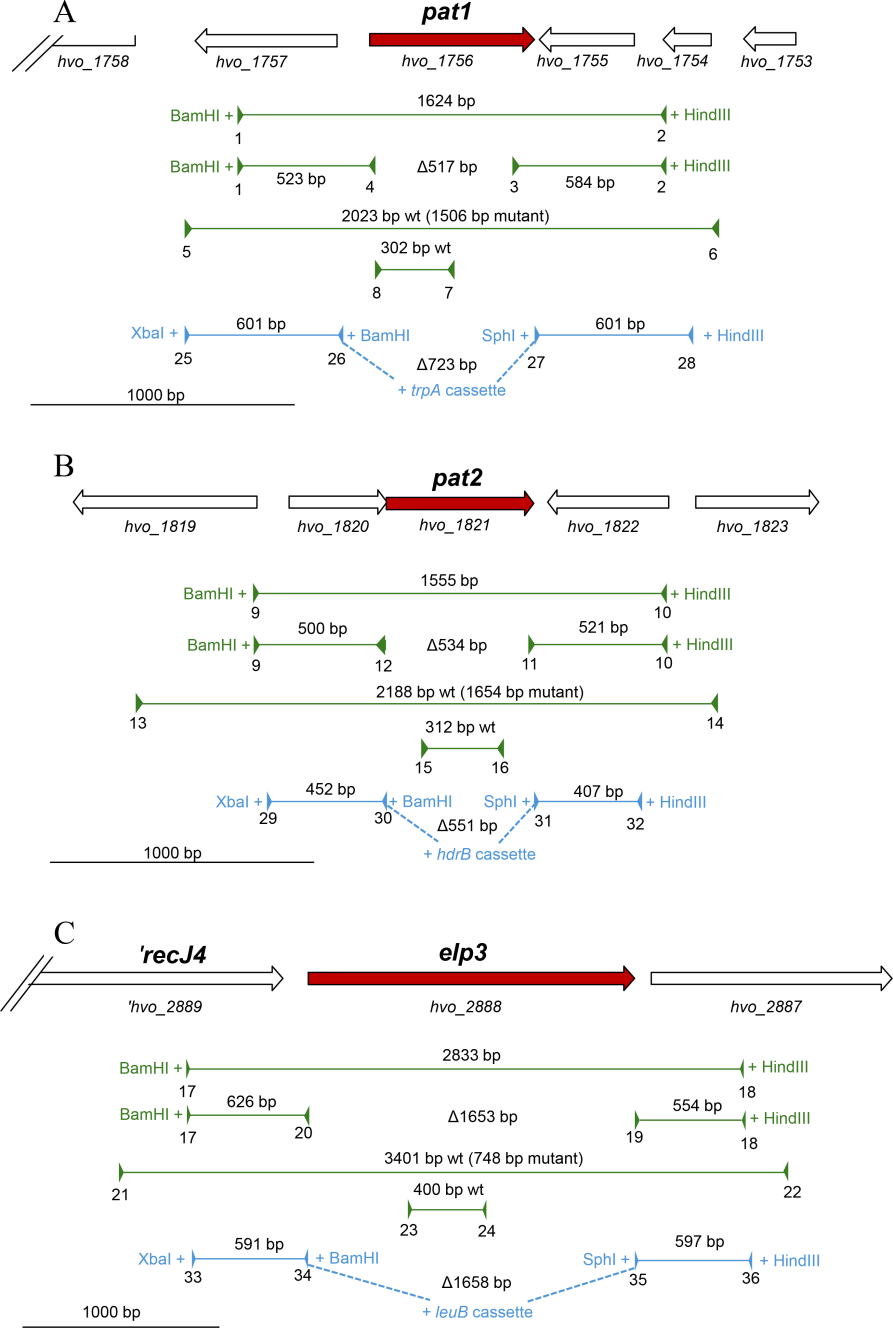
Strategies used to delete the *pat1*, *pat2*, and *elp3* genes in this study (green) compared to the past work by Altman-Price and Mevarech (15) (blue). The gene of interest is highlighted in red, and neighboring genes are highlighted in white. Plasmids and primers used for mutant strain construction and screening are listed in Tables 1 and 3, with corresponding primer numbers indicated. The pop-in/pop-out method (24) was employed in both studies with the following modifications as outlined below. In this study, pre-deletion plasmids were generated by inserting the gene of interest with 5′ and 3′ flanking regions into the BamHI and HindIII sites of vector pTA131. The resulting pre-deletion plasmids were used as DNA templates to generate the deletion plasmid by inverse PCR. Screening for the mutant strains was performed by PCR with external and internal primers. By contrast, Altman-Price and Mevarech generated the pre-deletion plasmids by inserting the 5′ and 3′ flanking regions of the gene of interest into the XbaI and HindIII sites of pTA131 and including BamHI and SphI sites in this process. The final mutagenesis plasmid was generated by inserting the selection markers *leuB*, *hdrB*, and *trpA* into the BamHI and SphI sites. The following clarifies the mutagenesis strategy for each gene targeted with primers indicated in parentheses: for *pat1* (**A**), a pre-deletion plasmid was generated by inserting a 1,624 bp *pat1* region (1 and 2) into the BamHI and HindIII sites of vector pTA131. The corresponding deletion plasmid was generated by inverse PCR (3 and 4), resulting in a 517 bp deletion (Δ*pat1_24-539_*). Screening for the deletion was performed using external primers (5 and 6) and internal primers (7 and 8). Altman-Price and Mevarech (15) generated the *pat1* pre-deletion plasmid by inserting two PCR products (25 and 26, 27 and 28) into the XbaI and HindIII sites of pTA131. The *trpA* cassette was inserted into the BamHI and SphI sites of this plasmid to generate the deletion plasmid that would result in a 723 bp deletion with a *trpA* insertion (Δ*intergenic_-99 to -1_* Δ*pat1_1-624_::trpA*). GenBank: *pat1* corresponds to CP001956.1: 1625273–1625896 complement; this study generated a deletion of CP001956.1: 1625357–1625873; Altman-Price and Mevarech generated a deletion of CP001956.1: 1625273–1625896. For *pat2* (**B**), a pre-deletion plasmid was generated by inserting a 1,555 bp *pat2* region (9 and 10) into the BamHI and HindIII sites of vector pTA131. The corresponding deletion plasmid was generated by inverse PCR (11 and 12), resulting in a 534 bp deletion (Δ*hvo_1820366_366-369_* Δ *pat2_1-534_*). Screening for the deletion was performed using external primers (13 and 14) and internal primers (15 and 16). Altman-Price and Mevarech generated the *pat2* pre-deletion plasmid by inserting two PCR products (29 and 30, 31 and 32) into the XbaI and HindIII sites of pTA131. The *hdrB* cassette was inserted into the BamHI and SphI sites of this plasmid to generate the deletion plasmid that would result in a 551 bp deletion and *hdrB* insertion (Δ*pat2_4-555_::hdrB*). GenBank: *pat2* corresponds to CP001956.1: 1683261–1683815; this study generated a deletion of CP001956.1: 1683261–1683794; Altman-Price and Mevarech generated a deletion of CP001956.1: 1625273–1625896. For *elp3* (**C**), the pre-deletion plasmid was generated by inserting a 2,833 bp *elp3* region (17 and 18) into the BamHI and HindIII sites of vector pTA131. The corresponding deletion plasmid was generated by inverse PCR (19 and 20), resulting in a 1653 bp deletion (Δ*elp3_6-1659_*). Screening for the deletion was performed using external primers (21 and 22) and internal primers (23 and 24). Altman-Price and Mevarech generated the *elp3* pre-deletion plasmid by inserting two PCR products (33 and 34, 35 and 36) into the XbaI and HindIII sites of pTA131. The *leuB* cassette was inserted into BamHI and SphI sites of this plasmid to generate the deletion plasmid that would result in a 1,658 bp deletion and *leuB* insertion (Δ*elp3_2-1659_::leuB*). GenBank: *elp3* corresponds to CP001956.1: 2726318–2727976 complement; this study generated a deletion of CP001956.1: 2726318–2727970; Altman-Price and Mevarech generated a deletion of CP001956.1: 2726318–2727970.

This study also highlights differences between the deletions observed in the current study and those reported in the previous study (15). To facilitate deletions, the previous study integrated selection markers on the genome to generate the Δ*pat2::hdrB* and Δ*elp3::leuB* mutations. The *hdrB* and *leuB* cassettes were placed between the *pat2* and *elp3* gene deletion regions, respectively. This strategy relies on genomic recombination events, with the markers adding more selective pressure, allowing for mutations to be more readily isolated (24). However, a disadvantage of this approach is the insertion of selection markers into the genome, which can potentially cause distal effects if placed in the middle of an operon or near small open reading frames near neighboring genes. The inverse *pat2* deletion plasmid used in the current study includes a 4-nucleotide deletion at the 5′ end of *hvo_1820*, encoding a universal stress protein A domain (UspA), thus generating the mutant Δ*hvo_1820366_366-369_* Δ*pat2_1-534_*. Additionally, the inverse deletion strategy for *pat2* leaves the last six amino acids of the C-terminus and the TGA stop codon intact (Fig. 4). The mutation of *hvo_1820* may have facilitated the deletion of *pat2* from KT10 by altering stress response pathways and regulation of *pat2*, potentially reducing the resistance to genomic modifications.

We were unsuccessful in generating Δ*elp3* Δ*pat1*Δ*pat2* triple mutants. The introduction of the *elp3* (pJAM4464) deletion plasmid into KT11 (H1207 Δ*pat1*Δ*pat2*) was achieved up to the final stages of counterselection, as the last three to four streaks resulted in unsuccessful isolation between wild type and targeted deletion. Similarly, attempts to introduce the smaller Δ*pat1* plasmid (pJAM4013) into KT18 (H1207 Δ*pat2*Δ*elp3*) and the Δ*pat2* plasmid (pJAM4014) into KT17 (H1207 Δ*pat1*Δ*elp3*) to generate a triple mutant faced challenges, with wild type being the predominant allele during PCR screening. The polyploid nature of *H. volcanii* (40) is suggested to be a potential factor in generating the triple mutant, resulting in incomplete genome deletion. Polyploidy, the presence of multiple copies of the genome, facilitates homologous recombination by providing wild-type templates for the repair of double-strand breaks (41–43). Polyploidy may also result in wild-type trace copies in the cell, if not all genome copies have been successfully mutated. Triple mutations could alternatively be attempted to be generated by introducing the gene of interest on a plasmid or under the control of a tryptophan-inducible promoter via conditional depletion, as seen successful for other *H. volcanii* genes (44, 45).

Phenotypic analysis revealed that the single or double deletions of lysine acetyltransferases impact the growth of *H. volcanii,* suggesting lysine acetylation plays a role in cellular fitness. While all three mutant strains (Δ*elp3*, Δ*pat1*Δ*elp3*, and Δ*pat2*Δ*elp3*) remained viable, each exhibited reduced growth when compared to the parent. Notably, the Δ*pat2*Δ*elp3* double mutant showed the most severe reduction in growth, suggesting that *elp3* and *pat2* have a synergistic role in promoting optimal growth. Although the double knock-out mutants of *pat1 elp3* and *pat2 elp3* are viable, this observation does not rule out an essential role for lysine acetylation in *H. volcanii*, as other acetyltransferases or chemical modifications may compensate for the loss of these genes. Lysine acetylation can be mediated by non-enzymatic mechanisms, as shown in other organisms (46). Moreover, the other uncharacterized acetyltransferase domain-containing proteins likely compensate for the loss of *pat1* and *pat2*, thereby maintaining acetylation. This point is supported by our previous study which observed lysine acetylation of the Δ*pat1*Δ*pat2* mutant by immunoblotting analysis (12).

Growth phenotypes associated with lysine acetyltransferase deletion mutants in *H. volcanii* are observable, with a more pronounced perturbation of growth on HY-rich vs ATCC974 media. The previous study (15) reported that the lysine acetyltransferase homolog deletion strains that could be generated (Δ*pat1*, Δ*pat2*, Δ*elp3*, Δ*pat1*Δ*pat2*, and Δ*pat1*Δ*elp3*) exhibited growth rates comparable to those of the H133 parent strain. In that study, *H. volcanii* was cultured in HY-rich medium. Here, we used ATCC974 to generate the mutant strains and then compared their growth on ATCC974 and HY-rich media to provide some insight into Δ*pat2*Δ*elp3* mutant viability. When comparing the two media (Table 6), the HY medium includes MnCl_₂_ as a trace element and Tris-HCl for pH buffering, while ATCC974 has a lower CaCl₂ concentration and includes tryptone, an additional source of peptides and amino acids. Moreover, ATCC974 medium contains a lower salt concentration, which offers advantages for autoclaving. These differences in the media may account for our ability to generate the Δ*pat2*Δ*elp3* mutant strain. Overall, the simultaneous double deletion of Δ*pat1*Δ*pat2*, Δ*pat1*Δ*elp3*, and Δ*pat2*Δ*elp3* is possible, providing understanding that these are not synthetic lethal gene pairs.

**TABLE 6:** Comparison of ATCC974 and HY-rich medium components per liter

Component	ATCC974 (pH 6.8)	Final concentration	HY-rich medium^*a*^	Final concentration
NaCl	125 g	2.13 M	150 g NaCl	2.57 M
Mg source	50 g MgCl_2_∙6H_2_O	245.9 mM	36.9 g MgSO_4_·7H_2_O	149.71 mM
K source	5 g K_2_SO_4_	28.69 mM	5 mL of 1 M KCl	5 mM
CaCl_2_	0.134 g	911.4 µM	5 mL of 10%CaCl_2_	3.4 mM
MnCl_2_	–^*b*^	–	1.8 mL of 75 mg/L MnCl_2_	1.07 µM
Yeast extract	5 g	–	5 g	–
Tryptone	5 g	–	–	–
Tris-HCl (pH 7.2)	–	–	50 mL of 1 M Tris-HCl	50 mM

^*a*^ %, wt/vol; HY-rich medium according to Altman-Price and Mevarech (15).

^*b*^ –, not added to medium.

## Data Availability

Sequencing data generated by this project were submitted to the National Center for Biotechnology Information (NCBI) Sequence Read Archive (SRA) and can be found under BioProject accession PRJNA1222392.

## SUPPLEMENTAL MATERIAL

Data Set 1 spectrum.01229-25-s0001.xlsx DNA sequence comparison of the *elp3 pat2* mutant and parent strains to the *H. volcanii* DS2 reference genome.

Fig. S1 spectrum.01229-25-s0002.tif HVO_1820 UspA domain protein models.

ASM does not own the copyrights to Supplemental Material that may be linked to, or accessed through, an article. The authors have granted ASM a non-exclusive, world-wide license to publish the Supplemental Material files. Please contact the corresponding author directly for reuse.
