# Supplementary figures and images for: Revisiting synthetic lethality of Gcn5-related N-acetyltransferase (GNAT) family mutations in Haloferax volcanii
Source: Microbiol Spectr. 2025 Jul 2;13(8):e01229-25. doi: 10.1128/spectrum.01229-25 (PMC12323606; doi:10.1128/spectrum.01229-25)

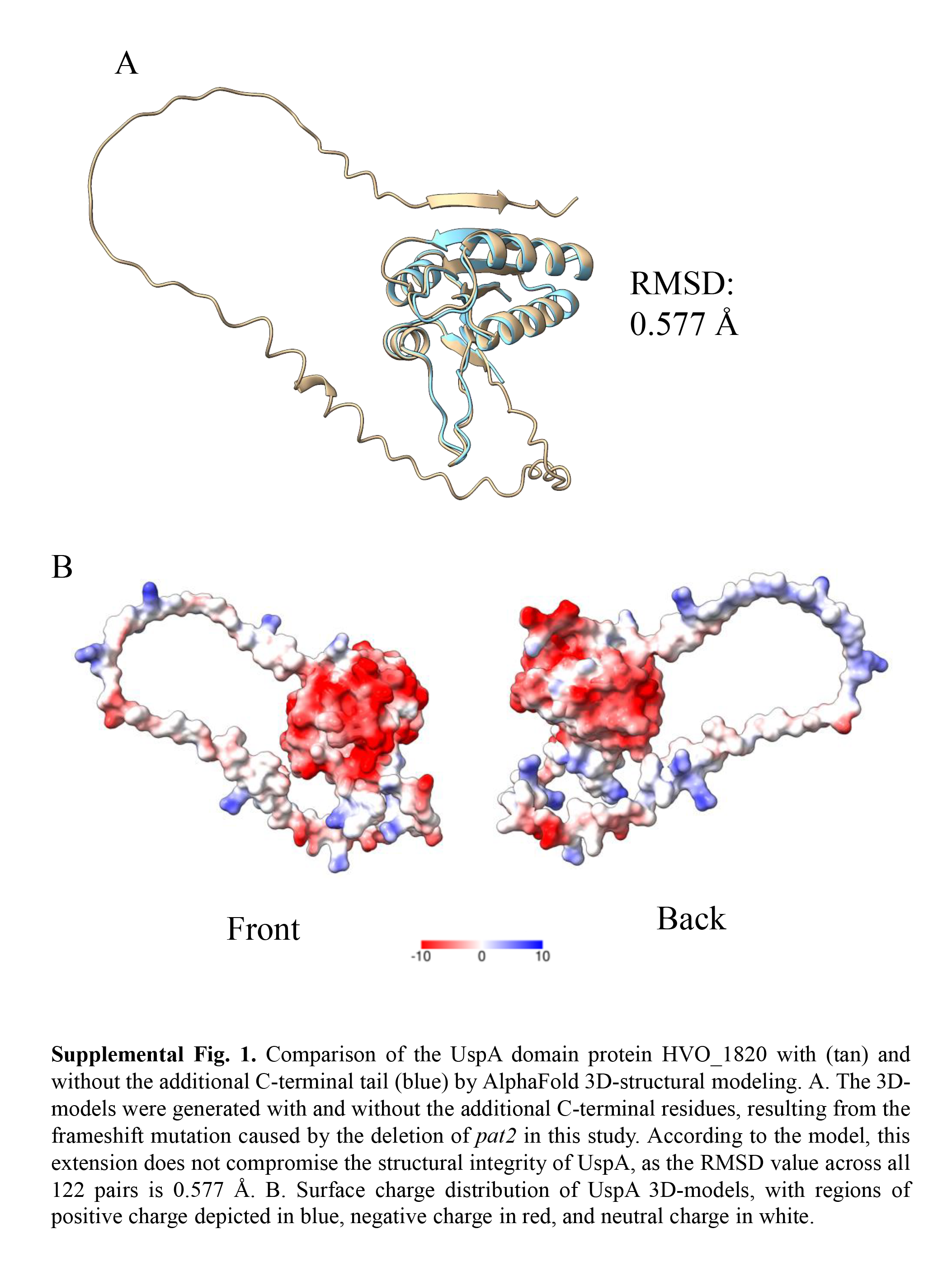

Supplement: Fig. S1 — HVO_1820 UspA domain protein models. [file spectrum.01229-25-s0002.tif]
